# The administration of *Fructus Schisandrae* attenuates dexamethasone-induced muscle atrophy in mice

**DOI:** 10.3892/ijmm.2015.2200

**Published:** 2015-05-04

**Authors:** JOO WAN KIM, SAE-KWANG KU, MIN HO HAN, KI YOUNG KIM, SUNG GOO KIM, GI-YOUNG KIM, HYE JIN HWANG, BYUNG WOO KIM, CHEOL MIN KIM, YUNG HYUN CHOI

**Affiliations:** 1 Research Institute, Bio-Port Korea INC, Marine Bio-industry Development Center, Busan 619-912, Republic of Korea; 2 Department of Anatomy and Histology, College of Korean Medicine, Daegu Haany University, Gyeongsan 712-715, Republic of Korea; 3 Department of Biochemistry, Dongeui University College of Korean Medicine, Busan 614-052, Republic of Korea; 4 Laboratory of Immunobiology, Department of Marine Life Sciences, Jeju National University, Jeju 690-756, Republic of Korea; 5 Anti-Aging Research Center and Blue-Bio Industry RIC, Dongeui University, Busan 614-714, Republic of Korea; 6 Departments of Food and Nutrition, College of Natural Sciences and Human Ecology, Dongeui University, Busan 614-714, Republic of Korea; 7 Departments of Life Science and Biotechnology, College of Natural Sciences and Human Ecology, Dongeui University, Busan 614-714, Republic of Korea; 8 Department of Biochemistry, Busan National University College of Medicine, Yangsan 626-870, Republic of Korea

**Keywords:** *Fructus Schisandrae*, muscle atrophy, dexamethasone, proteolysis, antioxidant effects

## Abstract

In the present study, we aimed to determine whether ethanol extracts of *Fructus Schisandrae* (FS), the dried fruit of *Schizandra chinensis* Baillon, mitigates the development of dexamethasone-induced muscle atrophy. Adult SPF/VAT outbred CrljOri:CD1 (ICR) mice were either treated with dexamethasone to induce muscle atrophy. Some mice were treated with various concentrations of FS or oxymetholone, a 17α-alkylated anabolic-androgenic steroid. Muscle thickness and weight, calf muscle strength, and serum creatine and creatine kinase (CK) levels were then measured. The administration of FS attenuated the decrease in calf thickness, gastrocnemius muscle thickness, muscle strength and weight, fiber diameter and serum lactate dehydrogenase levels in the gastrocnemius muscle bundles which was induced by dexamethasone in a dose-dependent manner. Treatment with FS also prevented the dexamethasone-induced increase in serum creatine and creatine kinase levels, histopathological muscle fiber microvacuolation and fibrosis, and the immunoreactivity of muscle fibers for nitrotyrosine, 4-hydroxynonenal, inducible nitric oxide synthase and myostatin. In addition, the destruction of the gastrocnemius antioxidant defense system was also inhibited by the administration of FS in a dose-dependent manner. FS downregulated the mRNA expression of atrogin-1 and muscle RING-finger protein-1 (involved in muscle protein degradation), myostatin (a potent negative regulator of muscle growth) and sirtuin 1 (a representative inhibitor of muscle regeneration), but upregulated the mRNA expression of phosphatidylinositol 3-kinase, Akt1, adenosine A1 receptor and transient receptor potential cation channel subfamily V member 4, involved in muscle growth and the activation of protein synthesis. The overall effects of treatment with 500 mg/kg FS were comparable to those observed following treatment with 50 mg/kg oxymetholone. The results from the present study support the hypothesis that FS has a favorable ameliorating effect on muscle atrophy induced by dexamethasone, by exerting anti-inflammatory and antioxidant effects on muscle fibers, which may be due to an increase in protein synthesis and a decrease in protein degradation.

## Introduction

A progressive loss of muscle mass and strength, known as sarcopenia, represents an important risk factor for disability and mortality. The loss of skeletal muscle mass has a profound effect on the daily life of patients, particularly on physical activity. The resulting decrease in physical activity induces further skeletal muscle atrophy, leading to a vicious cycle of atrophic processes (1,2). The main factors that cause muscle atrophy are denervation, musculoskeletal injury, joint immobilization, ligament and joint injury, joint inflammation, prolonged bed rest, glucocorticoid (GLU) treatment, sepsis, cancer and aging (3,4). Atrophy begins with a reduction in muscle tension, which is reflected in both a decrease in synthesis and an increase in protein degradation (5). Four systems of proteolytic degradation are involved in muscle atrophy: the lysosomal protease system (cathepsin), calpain calcium-dependent signaling, caspase signaling and the ubiquitin-proteasome system (5,6). Oxidative stress has also been well-established as an important inducer of muscle atrophy in both disuse and muscle catabolic cachexia (7).

Various animal models of skeletal muscle atrophy have been used in research, including unloading (8), immobilization (9), starvation (10), denervation (11) and the administration of GLU (12). Among these, the administration of high concentrations of dexamethasone (a representative GLU) causes catabolic changes in skeletal muscle, mainly due to the stimulation of muscle proteolysis. This GLU-induced protein degradation is mainly mediated by the activation of the ubiquitin-proteasome and lysosomal pathways (13,14). In particular, the muscle-specific E3-ligases, atrogin-1 and muscle RING-finger protein-1 (MuRF1), and the lysosomal enzyme, cathepsin L, are highly stimulated by GLUs (15,16). The upregulation of myostatin, a member of the transforming growth factor (TGF)-β family, is also an important negative regulator of skeletal muscle mass that is involved in GLU-induced catabolic muscle atrophy (17). These findings suggest that GLU-induced skeletal muscle atrophy may serve as a useful and rapid animal model for screening agents that can prevent abnormal catabolic muscle atrophy (18,19).

The dried fruit of *Schizandra chinensis* Baillon (*S. chinensis*), *Fructus Schisandrae* (FS), is a well-known traditional herb used for pharmacological purposes in Asian countries (e.g., Korea, China and Japan) and in Russia to increase physical working capacity and for its stress-protective effects against aseptic inflammation and heavy metal intoxication. It also has beneficial effects on the central nervous, sympathetic nervous, endocrine, immune, respiratory, cardiovascular and gastrointestinal systems. It inhibits the development of experimental atherosclerosis, controls blood sugar and acid-base balance, and regulates uterus myotonic activity (20,21). In addition, recent studies have suggested that FS exerts favorable effects on diabetes and related complications (22–25) due to its smooth muscle relaxant effects (26,27). However, the effectiveness of FS administration in the prevention of GLU-induced muscle atrophy remains unclear.

The aim of the present study was to investigate the effects of the administration of FS ethanol extracts on dexamethasone-induced skeletal muscle atrophy *in vivo*. In addition, we evaluated the molecular mechanisms involved in dexamethasone-induced muscle atrophy and the inhibitory effects of FS on these molecular events, in an aim to determine whether the administration of FS has therapeutic value as a treatment for GLU-induced muscle atrophy.

## Materials and methods

### Test materials

The fruits of *S. chinensis* were collected from an area around the city of Mungyeong (Gyeongsangbuk-do, Korea) and washed 3 times with tap water before being stored at −20°C. The frozen samples were lyophilized and homogenized using a grinder prior to extraction. The materials were extracted with 20% ethanol (FS) at room temperature for 24 h. The extract solution was filtered and concentrated using a rotary vacuum evaporator (Buchi Rotavapor R-144, Büchi Labortechnik AG, Flawil, Switzerland). Oxymetholone [17β-hydroxy-2-(hydroxymethylene)-17-methyl-5α-androstane-3-one; Celltrion Pharm Inc., Jincheon, Korea], which is an orally active 17α-alkylated anabolic-androgenic steroid, was used as the reference drug. Oxymetholone was dissolved at 5 mg/ml in distilled water and FS was dissolved at 50 mg/ml in distilled water, and dexamethasone (Sigma-Aldrich Chemical Co., St. Louis, MO, USA) was dissolved at 50 mg/ml in distilled water. They were stored in at 4°C and diluted with medium to the desired concentration prior to use.

### Animals and experimental design

Adult SPF/VAF outbred CrljOri:CD1 (ICR) mice (OrientBio Inc., Seungnam, Korea) were used in the experiments in the present study. The animals were maintained under controlled environmental conditions under a 12 h/12 h light/dark cycle and were allowed *ad libitum* access to water and a standard laboratory diet. Six groups of 8 mice in each group [i) the intact vehicle control, ii) the dexamethasone control group, iii) the oxymetholone-treated group and the FS-treated groups: iv) FS 125 mg/kg-treated group, v) FS 250 mg/kg-treated group and SF 500 mg/kg treated group] were created in which the mice were selected based on body weight (35.76±1.32 g; range, 33.40–38.50 g) and calf thickness (3.15±0.14 mm; range, 2.84–3.42 mm) after 8 days of acclimatization. Three different concentrations of FS (125, 250 and 500 mg/kg body mass) were orally administered, once a day, for 24 days; treatment with FS was initiated 2 weeks before dexamethasone treatment, and 50 mg/kg of oxymetholone were also orally administered in the same time period as FS administration. In this study, muscle atrophy was induced by a subcutaneous injection of dexamethasone (1 mg/kg), once a day for 10 days according to a previously established method (14). An equal volume of distilled water was orally administered to the mice in the intact vehicle control and dexamethasone control groups, instead of FS or oxymetholone, and saline was subcutaneously injected into the mice in the intact vehicle control gorup instead of dexamethasone. The dosage of oxymetholone was selected as 50 mg/kg based on a previous efficacy test in mice (28). This experiment was conducted according to the international regulations of the usage and welfare of laboratory animals, and approved by the Institutional Animal Care and Use Committee of Daegu Haany University (Gyeongsan, Korea) (Approval no. DHU2014-003).

### Measurement of body weight, calf thickness and gastrocnemius muscle thickness

The body weights of all the mice and the thickness (mm/mouse) of the left hind calf were measured at 1 day before, and on days 0, 1, 7, 14, 19, 23 and 24 of the test material administration using an automatic electronic balance machine (Precisa Instruments, Dietikon, Switzerland) and an electronic digital caliper (Mytutoyo, Tokyo, Japan), respectively. The gastrocnemius muscle thickness of the left hind limb was also measured using the same method following the exposure of the muscle at sacrifice to reduce the differences from surrounding tissues, and the changes in calf thickness during the 14 days prior to the administration of the test material, during the 10 days of administration, and for the total 24 days of administration were additionally calculated to reduce individual differences. Mice were sacrificed by exsanguination under anesthesia with a 25 mg/kg intraperitoneal injection of zoletil (Zoletil 50^®^; Virbac, Nice, France).

### Measurement of gastrocnemius muscle weight

After measuring gastrocnemius muscle thickness at sacrifice, the gastrocnemius muscle masses were carefully separated from the tibia and fibula bones. The weights of individual gastrocnemius muscle masses were measured at the g levels (absolute wet weight) using an automatic electronic balance machine, and the relative weight (% of body weight) was also calculated to reduce the differences from individual body weights, using body weight at sacrifice and absolute weight, as follows: relative muscle weight (% of body weight) = [(absolute organ weight/body weight at sacrifice) ×100].

### Measurement of calf muscle strength

One hour after the final (24th) administration of the vehicle, oxymetholone or FS (10 days after the initial dexamethasone treatment), the calf muscle strength of individual mice was measured as tensile strength (N) using a computerized testing machine (SV-H1000, Japan Instrumentation System Co., Tokyo, Japan). Briefly, the animals were restrained in the machine using two separated 1-0 silk suture ties on the left ankle and chest, and the peak tensile loads were recorded as calf muscle strength, at knee angles of 0° (10–20 mm distances).

### Serum biochemistry

Sera for blood biochemistry were obtained on the day of necropsy by centrifuging the blood samples in a separation tube at 3,000 rpm for 10 min. Serum creatine and creatine kinase (CK) levels were measured using an autoanalyzer (Hemagen Analyst, Hemagen Diagnostics, Columbia, MD, USA) and lactate dehydrogenase (LDH) levels were measured on an automated serum biochemistry analyzer (SP-4410, Spotochem, Tokyo, Japan).

### Antioxidant defense systems

After measuring the muscle weight, the gastrocnemius muscles were separated for the assessment of malondialdehyde (MDA), reactive oxygen species (ROS) and glutathione (GSH) contents, as well as catalase (CAT) and superoxide dismutase (SOD) enzyme activity in individual muscle. The separated gastrocnemius muscles were weighed and homogenized in ice-cold 0.01 M Tris-HCl (pH 7.4), and then centrifuged, at 12,000 g for 15 min, as previously described by Del Rio *et al* (29). The concentrations of muscle lipid peroxides (nmoles MDA/g tissue) were determined by estimating MDA levels using the thiobarbituric acid test at an absorbance of 525 nm with a UV/Vis spectrometer (Optizen POP Mecasys, Daejeon, Korea), as previoulsy described (30). Total protein levels were measured using a previously described method (31) using bovine serum albumin (Invitrogen, Carlsbad, CA, USA) as a standard. Skeletal muscles were homogenized and the ROS levels were analyzed using 2′,7′-dichlorofluorescein diacetate fluorescent dye as a probe and fluorescence density measurement at 490/520 nm according to the manufacturer’s instructions (ROS assay kit; Abcam, Cambridge, MN, USA). The measured optical density values [relative fluorescence unit (RFU)] were corrected by the protein concentrations of the samples and were expressed as RFU/*μ*g protein, as previoulsy described (32). The prepared homogenates were mixed with 0.1 ml of 25% trichloroacetic acid (Merck, San Francisco, CA, USA), and then centrifuged at 4,200 rpm for 40 min at 4°C. GSH contents (mg/g tissue) were measured at absorbance of 412 nm using 2-nitrobenzoic acid (Sigma-Aldrich Chemical Co.), as previously described (33). The decomposition of H_2_O_2_ in the presence of CAT was followed by measuring the absorbance at 240 nm, as previously described (34). CAT activity was defined as the amount of enzyme required to decompose 1 nM of H_2_O_2_ per minute, at 25°C and pH 7.8. The results were expressed as U/mg protein. Measurements of SOD activity were made according the method described in the study by Sun *et al* (35). The estimation of SOD activity was based on the generation of superoxide radicals produced by xanthine and xanthine oxidase, which react with nitrotetrazolium blue to form a formazan dye. SOD activity (U/mg protein) was then measured at 560 nm as the degree of inhibition of this reaction. One unit of SOD enzymatic activity is equal to the amount of enzyme that diminishes the initial absorbance of nitroblue tetrazolium by 50% during 1 min.

### Reverse transcription-quantitative polymerase chain reaction (RT-qPCR)

RNA was extracted using TRIzol reagent (Invitrogen), according to the manufacturer’s instructions. The RNA concentrations and quality were determined using a CFX96™ Real-Time PCR detection system (Bio-Rad, Hercules, CA, USA). Contaminated DNA was removed by treating the samples with recombinant DNase I (DNA-free; Ambion, Austin, TX, USA). RNA was reverse transcribed using the reagent High-Capacity cDNA Reverse Transcription kit (Applied Biosystems, Foster City, CA, USA) according to the manufacturer’s instructions. The internal control was 18S ribosomal RNA. The sequences of the PCR oligonucleotide primers are listed in Table I.

### Histopathological anlaysis

The samples of gastrocnemius muscle were separated and fixed in 10% neutral-buffered formalin, embedded in paraffin, sectioned (3–4 *μ*m) and then stained with hematoxylin and eosin (H&E) for general histopathological anlaysis, as previously described (36) or with Sirius red for detecting collagen fibers, as previously described (37). The histopathological profiles of each sample were then determined by light microscopy observation (Nikkon, Japan). More detailed changes in the gastrocnemius muscle samples were obtained by calculating the mean muscle fiber diameters (*μ*m/fiber) and the regions occupied by collagen fibers (%/mm^2^ of muscle bundles) in the muscle bundles for general histomorphometrical analysis using an automated image analyzer (iSolution FL version 9.1, IMT i-solution Inc., Quebec, Canada), according to previously described methods (17,36) with minor modifications.

### Immunohistochemistry

The sections were deparaffinized and pre-treated for citrate buffer antigen (epitope) retrieval, as previously described (38). Briefly, a staining dish containing 10 mM citrate buffer (pH 6.0) was heated in a water bath to a temperature of 95–100°C. The slides were immersed in the staining dish, loosely covered, incubated for 20 min and then left to cool for 20 min at room temperature. Following epitope retrieval, the sections were then immunostained using avidin-biotin complex (ABC) methods for nitrotyrosine, 4-hydroxynonenal (4-HNE), inducible nitric oxide synthase (iNOS), and myostatin (Table II), according to a previously described method (39). The cells or muscle fibers occupying >20% of immunoreactivities, the density, of each antiserum for nitrotyrosine, 4-HNE, iNOS, cyclooxygenase-2 (COX-2), tumor necrosis factor (TNF)-α and myostatin as compared with the intact muscles, were regarded as positive, and the mean number of nitrotyrosine, 4-HNE, iNOS and myostatin-immunoreactive fibers or COX-2 and TNF-α- immunoreactive cells dispersed in the mm^2^ of muscle bundles was counted using an automated image analysis process as per previously established methods (40,41) with minor modifications. A histopathologist blinded to the group distribution performed the analysis.

### Statistical analyses

Multiple comparison tests were conducted for the different groups. Variance homogeneity was examined using the Levene test (42). If the Levene test indicated no significant deviations from variance homogeneity, the obtained data were analyzed by one-way ANOVA followed by a least-significant differences multi-comparison (LSD) test to determine which pairs of group comparisons were significantly different. When significant deviations from variance homogeneity were observed with the Levene test, a non-parametric comparison test (Kruskal-Wallis H test) was conducted. When a significant difference was observed in the Kruskal-Wallis H test, the Mann-Whitney U (MW) test was conducted to determine which specific pairs of the group comparison were significantly different. Statistical analyses were conducted using SPSS software for Windows (Release 14K, SPSS Inc., USA), as previously described (43).

## Results

### The administration of FS mitigates the dexamethasone-induced loss of body weight and calf thickness

**Figure 1:**
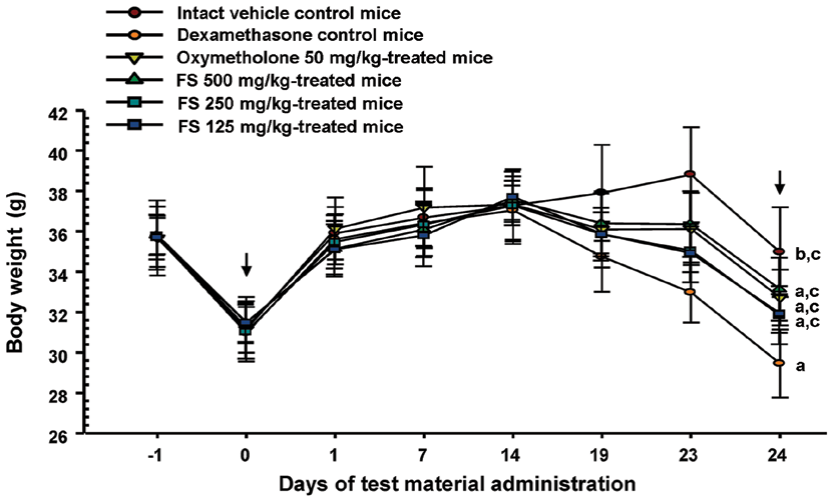
Changes in body weight in mice with dexamethasone-induced muscle atrophy. The body weight of each mouse was measured at 1 day before, and on days 0, 1, 7, 14, 19, 23 and 24 of the test material administration as described in the Materials and methods. Values are expressed as the means ± SD of 8 mice. Days -1 and 24 indicate 1 day before the start of test material administration and at sacrifice, respectively. Zero indicates the start of the test material administration, at 2 weeks before the initial dexamethasone treatment. All animals were fasted overnight before the first test material administration and before sacrifice (arrows). ^a^p<0.01 and ^b^p<0.05 as compared with the intact vehicle control by the LSD test. ^c^p<0.01 as compared with the dexamethasone control by the LSD test. FS, *Fructus Schisandrae.*

**Figure 2:**
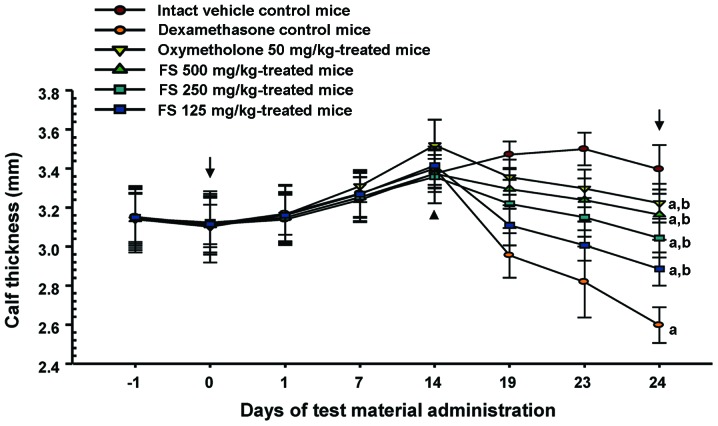
Changes in calf thickness in mice with dexamethasone-induced muscle atrophy. The thickness of the left hind calf was measured at 1 day before and on days 0, 1, 7, 14, 19, 23 and 24 of the test material administration, as described in the Materials and methods. Values are expressed as the means ± SD of 8 mice. Days -1 and 24 indicate 1 day before the start of test material administration and at sacrifice, respectively. Zero indicates the start of the test material administration, at 2 weeks before the initial dexamethasone treatment. All animals were fasted overnight before the first test material administration and before sacrifice (arrows). ^a^p<0.01 as compared with the intact vehicle control by the LSD test. ^b^p<0.01 as compared with the dexamethasone control by the LSD test. FS, *Fructus Schisandrae.*

As shown in Fig. 1, the mice in the dexamethasone control group presented with progressive body weight loss throughout the study as compared with the intact vehicle control group. However, this decrease in body weight was significantly inhibited by treatment with oxymetholone and all 3 concentrations of FS at the final (24th) administration and at sacrifice. As was expected, treatment with dexamethasone caused a significant decrease in calf thickness compared with the intact vehicle controls from day 19 after the administration of the first test substance, the day of the 5th dexamethasone treatment until sacrifice (Fig. 2). This decrease in calf thickness was also significantly inhibited in a dose-dependent manner by treatment with FS compared with the dexamethasone controls. In this study, the oxymetholone-treated mice showed a significant increase in calf thickness from the 14th treatment compared to the mice in the intact vehicle control and the dexamethasone control groups.

### Effects of the administration of FS on the dexamethasone-induced loss of gastrocnemius muscle thickness and weight

**Figure 3:**
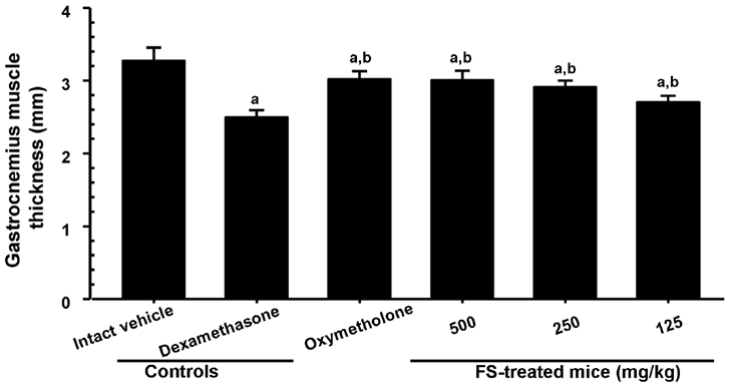
Changes in gastrocnemius muscle thickness in mice with dexamethasone-induced muscle atrophy. The gastrocnemius muscle thickness of the left hind limb was also measured after exposing the muscles at sacrifice, as described in the Materials and methods. Values are expressed as the means ± SD of 8 mice. ^a^p<0.01 as compared with the intact vehicle control by the LSD test. ^b^p<0.01 as compared with the dexamethasone control by the LSD test. FS, *Fructus Schisandrae.*

**Figure 4:**
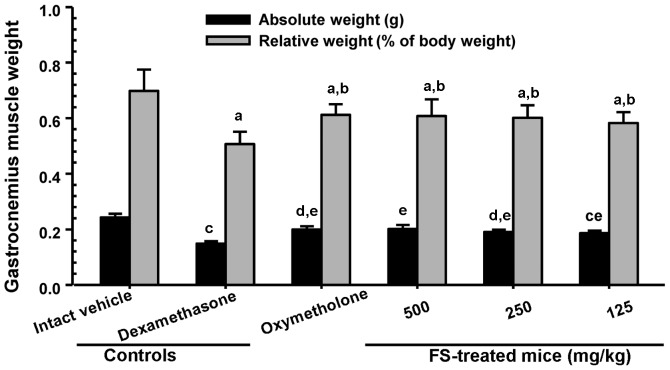
Changes in the gastrocnemius muscle weight in mice with dexamethasone-induced muscle atrophy. After measuringgastrocnemius muscle thickness at sacrifice, the gastrocnemius muscle mass was carefully separated from the tibia and fibula bones and weighed (absolute wet-weight). Values are expressed as the means ± SD of 8 mice. ^a^p<0.01 as compared with the intact vehicle control by the LSD test. ^b^p<0.01 as compared with the dexamethasone control by the LSD test. ^c^p<0.01 and ^d^p<0.05 as compared with the intact vehicle control by the MW test. ^e^p<0.01 as compared with the dexamethasone control by the MW test. FS, *Fructus Schisandrae*.

To investigate muscle loss following treatment, gastrocnemius muscle thickness and weight were measured immediately after biopsy. As shown in Fig. 3, a significant decrease in gastrocnemius muscle thickness after the exposure of the muscle (through skin removal) was observed in the mice in the dexamethasone control group compared with the mice in the intact vehicle control group. A significant increase in gastrocnemius muscle thickness was observed in the mice administered oxymetholone and all 3 different concentrations of FS compared with the dexamethasone controls. A significant decrease in absolute wet-weight and the relative weight of the gastrocnemius muscle were also observed in the mice in the dexamethasone control group compared with the mice in the intact vehicle control group (Fig. 4). The adminsitration of FS effectively prevented the dexamethasone-induced decrease in muscle weight compared with the dexamethasone controls (Fig. 4).

### The administration of FS inhibits the dexamethasone-induced decrease in muscle strength

**Figure 5:**
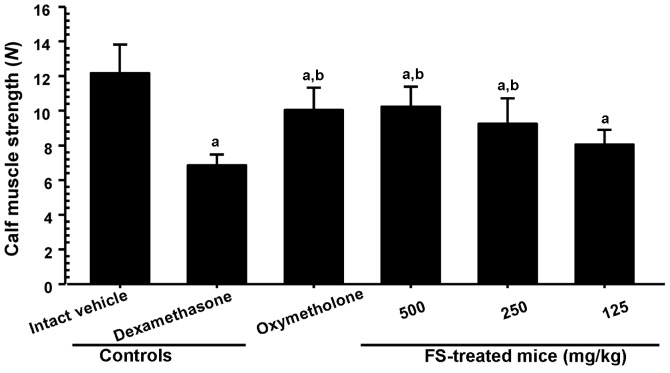
Changes in calf muscle strength in mice with dexamethasone-induced muscle atrophy. One hour after the final (24th) administration of the vehicle (distilled water), oxymetholone, or FS, the calf muscle strength of individual mice was measured as described in the Materials and methods. Values are expressed as the means ± SD of 8 mice (N, Newton). ^a^p<0.01 as compared with the intact vehicle control by the LSD test. ^b^p<0.01 as compared with the dexamethasone control by the LSD test. FS, *Fructus Schisandrae*.

Since reduced absolute muscle strength may reflect the loss of muscle mass (1,2), we analyzed the effects of the administration of FS on calf muscle strength. As was expected, treatment with dexamethasone resulted in a significant decrease in the tensile strength of the calf muscles when compared with the intact vehicle control mice (Fig. 5). However, a significant increase in calf muscle strength was observed in the mice administered oxymetholone and 500 and 250 mg/kg FS compared with the dexamethasone controls. In addition, the mice treated with 125 mg/kg FS also showed an increase in calf muscle strength compared with the dexamethasone controls, although this difference was not statistically significant (when compared with the dexamethasone controls).

### Effects of the administration of FS on serum biochemistry

Since the appearance of CK associated with the decrease in LDH levels in blood serum is considered a surrogate marker of muscle damage (3,4), we measured the levels of serum creatine, CK and LDH. A significant increase in serum creatine and CK levels, and a decrease in serum LDH levels were observed in the mice in the dexamethasone control group compared with the mice in the intact vehicle control group; however, the administration of oxymetholone and FS effectively attenuated the dexamethasone-induced increase in creatine and CK levels. Oxymetholone and FS also significantly increased the serum LDH levels (Table III).

### Effects of the administration of FS on gastrocnemius muscle antioxidant defense systems

Oxidative stress due to greater levels of ROS production than those normally neutralized by intracellular antioxidant defenses has recently gained much attention for its possible involvement in muscle disuse atrophy (7). As shown in Table IV, a significant increase in muscle lipid peroxidation (elevation of MDA levels) and ROS contents were observed in the mice in the dexamethasone control group compared with the mice in the intact vehicle control group. These elevated levels of MDA and ROS were significantly decreased by treatment with FS in a dose-dependent manner. In addition, the elevated lipid peroxide and ROS levels in the oxymetholone-treated mice were also significantly decreased compared with the dexamethasone control mice. In addition, a significant decrease in endogenous antioxidant (GSH) levels and antioxidative enzyme (SOD and CAT) activity were detected in the dexamethasone controls compared with the intact vehicle controls, and this decrease was significantly inhibited by 24 days of continuous oral treatment with oxymetholone or FS.

### Effects of the administration of FS on mRNA expression levels in gastrocnemius muscle

To assess the mechanisms responsible for the inhibitory effects of FS on dexamethasone-induced muscle atrophy, we measured the levels of genes involved in muscle growth, regeneration and the activation of protein synthesis. As presented in Table V, a significant increase in the expression of gastrocnemius muscle atrogin-1, MuRF1, myostatin and sirtuin 1 (SIRT1) mRNA expression was observed in the dexamethasone controls compared with the intact vehicle controls. This elevated expression was significantly decreased by treatment with FS in a dose-dependent manner. These expression levels in the oxymetholone-treated mice also showed a significant decrease compared with the mice in the dexamethasone control group. A significant decrease in the mRNA expression of phosphatidylinositol 3-kinase (PI3K), Akt1, adenosine A1 receptor (A1R) and transient receptor potential cation cannel subfamily V member 4 (TRPV4) was observed in the dexamethasone controls compared with the intact vehicle controls. A significant increase in these expression levels was observed with all 3 FS concentrations used in a dose-dependent manner compared with the dexamethasone controls.

### Histopathological and immunohistochemical anlaysis of the effects of the administration of FS on gastrocnemius muscle

**Figure 6:**
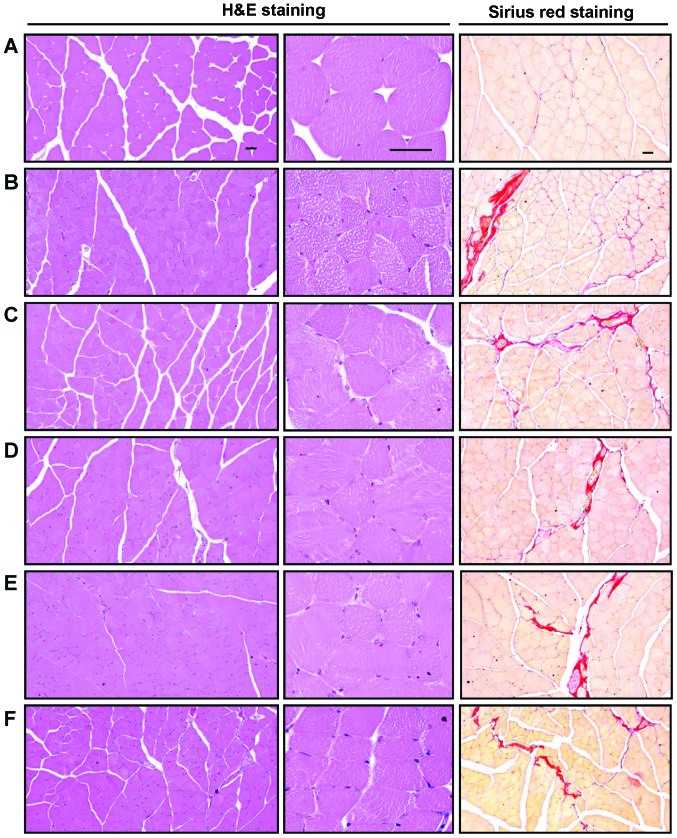
Representative gastrocnemius muscle histological images, taken from mice in the intact vehicle control or dexamethasone control groups. Samples from gastrocnemius muscle were separated and fixed in 10% neutral buffered formalin, then embedded in paraffin, sectioned and stained with H&E for general histo-pathological analysis or with Sirius red for the detection of collagen fibers, and the histopathological profiles of each sample were determined by light microscopy. (A) Intact vehicle control, (B) dexamethasone control, (C) 50 mg/kg oxymetholone-treated mice, (D) 500 mg/kg FS orally-treated mice, (E) 250 mg/kg FS mg/kg orally-treated mice, (F) 125 mg/kg FS orally-treated mice. Scale bars, 40 *μ*m. FS, *Fructus Schisandrae.*

In the histopathological analysis of muscle atrophy, we observed that marked and classic catabolic muscle atrophic changes, including diminishing muscle fibers, microvacuolation and focal fibrosis in the muscle bundles were induced by treatment with dexamethasone, as well as a significant decrease in the mean muscle fiber diameters. An increase in the number of regions occupied by collagen fibers in muscle bundles was detected in the mice in the dexamethasone control group compared with the mice in the intact vehicle control group (Table VI and Fig. 6). These atrophic changes were markedly decreased by treatment with FS in a dose-dependent manner. The muscle atrophic changes were also significantly inhibited in the oxymetholone-treated mice compared with the dexamethasone control mice. Moreover, a marked and significant increase in nitrotyrosine-, 4-HNE- and iNOS-immunoreactive fibers was also observed in the mice in the dexamethasone control group; however, FS normalized these dexamethasone-related changes in a dose-dependent manner (Table VI). Oxymetholone also significantly decreased the nitrotyrosine-, 4-HNE- and iNOS-positive muscle fiber numbers compared with those obseved in the mice in the dexamethasone control group. Furthermore, the numbers of immunoreactive fibers stained for myostatin, a potent negative regulator of muscle growth, were significantly increased in the mice in the dexamethasone control group. However, FS significantly normalized these changes and oxymetholone also significantly decreased the numbers of myostatin-positive muscle fibers compared with those observed in the mice in the dexamethasone control group.

## Discussion

Catabolic muscle atrophy induced by GLU is characterized by a reduction in protein content, the loss of organelles, cytoplasm, fiber diameter, muscle strength and resistance to fatigue (14,17). Millions of individuals take GLUs as chronic therapy for the treatment of diseases, such as rheumatoid arthritis, asthma, organ transplants and primary or secondary adrenal insufficiency (44). Common side-effects of GLUs include insomnia, nervousness, gastrointestinal upset, arthralgias, immunosuppression, edema and myopathy (45). With over 50 years of use, GLUs are one of the common medications known to cause myopathy, particularly when used for prolonged periods ofm time at high concentrations (46). The incidence of muscle weakness and myopathy can be as high as 50% in persons receiving long-term GLU therapy (47,48). The characteristics of myopathy include muscle atrophy and weakness, insulin resistance, oxidative stress and mitochondrial dysfunction. Steroid-induced myopathy is proximal and symmetrical and may involve both the upper and lower extremities. Steroid myopathy is more commonly associated with the use of fluorinated steroids, such as dexamethasone, ;betamethasone and triamcinolone, but can also be caused by non-fluorinated steroids, such as prednisolone and hydrocortisone (49). In the present study, the beneficial effects of FS on skeletal muscle were observed in the mice with GLU (dexamethasone)-induced catabolic muscle atrophy.

All mice used in this study as intact vehicle controls presented with a normal body weight throughout the experimental period, which was within the normal range for age-matched normal reference mice (50). No changes in body weight related to treatment with the test materials were observed compared with the mice in the intact vehicle control or dexamethasone control groups, whereas a signifi-cant decrease in body weight was observed in the mice in the dexamethasone control group compared with the mice in the intact vehicle control beginning 5 days after the initial dexamethasone treatment until sacrifice (Fig. 1). This decrease in body weight induced by treatment with dexamethasone was considered to be due to cachexia-related changes resulting from the potent catabolic effects of dexamethasone itself (51,52), and the increase in body weight detected with all 3 FS concentrations may be related at least in part to the well-documented immuno modulatory effects of FS (53,54). Generally, animals with enhanced immune systems show relatively good growth patterns (55,56). In addition, oxymetholone, a representative 17α-alkylated anabolic-androgenic steroid (28), may have inhibited the catabolic cachexia-related decrease in body weight induced by GLU treatment through its potent anabolic effects (57,58).

High concentrations of GLU can induce catabolic muscle atrophy characterized by a reduction and degradation of the protein content, a decrease in the fiber diameter, a reduction in muscle strength and poor resistance to fatigue (17,44). Accordingly, in this study, a decrease in calf thicknesses was noted from 5 days after the initial dexamethasone treatment, and a decrease in calf muscle strength, gastrocnemius muscle thickness and body weight at sacrifice was also observed in the dexamethasone treated-mice as a result of catabolic muscle atrophy (Figs. 2–5). Treatment with oral FS and 50 mg/kg oxymetholone resulted in similar responses, thus providing direct evidence that both FS and oxymetholone ameliorated the dexamethasone-induced calf muscle atrophic changes. In this study, treatment with 500 mg/kg FS showed similar favorable effects on calf muscle preservation to those observed following treatment with 50 mg/kg oxymetholone.

Creatine is a nitrogenous organic acid that occurs naturally in vertebrates and helps to supply energy to all cells in the body, primarily muscle. Creatine synthesis occurs in the liver and kidneys, but not in muscle, which has no creatine synthesis capacity, and creatine is accumulated in muscle against a concentration gradient through specific active transport from plasma (59). An estimated 98% of total-body creatine is found in skeletal muscle. The creatine content in skeletal muscle is relatively constant (60). Creatine is metabolized to its non-ionic cyclic derivative creatinine at a constant rate of over 1.7% per day (61) by a non-enzymatic hydrolytic cyclization that is irreversible *in vivo* (62). Creatinine rapidly diffuses from muscle into the plasma and urine with no re-uptake into muscle (59). It is not significantly otherwise metabolized, and its excretion under steady-state conditions therefore equals creatinine production and is proportional to the total-body creatine pool size and skeletal muscle mass (59,63). Plasma creatine levels can therefore be used as a valuable serum biochemistry marker indicating skeletal muscle damage, activity, or amounts (64,65). In the present study, a marked increase in serum creatine levels was observed along with GLU-related catabolic muscle atrophic changes, as previously demonstrated (13); however, treatment with FS significantly inhibited this increase in a dose-dependent manner (Table III). Treatment with FS at 500 mg/kg in particular, showed inhibitory effects on serum creatine levels comparable to those observed with 50 mg/kg oxymetholone, again suggesting that FS has favorable effects on muscle preservation against muscle atrophy induced by dexamethasone.

LDH is of medical significance as it is found extensively in body tissue, such as blood cells and heart muscle, and CK is an enzyme expressed by various tissues and cell types. CK cata-lyzes the conversion of creatine and consumes adenosine. Since these factors are released during tissue damage, they are serum markers of common injury and disease, particularly muscle damage (66,67). They are also markedly elevated in animals with disuse muscle atrophy (68). In a previous study, muscle atrophy induced by treatment with dexamethasone resulted in a marked elevation in serum CK levels (69), but in another study, serum LDH levels were generally decreased due to a reduction in physiological activity, i.e., reduced contractions of skeletal muscle fibers (70). A significant elevation in serum CK levels indicating muscle damage and a decrease in serum LDH levels suggesting a reduction in muscle activity were also observed in the mice in the dexamethasone control group in the present study. A similar concentration-dependent decrease in serum CK levels and an increase in serum LDH levels were observed in the FS- and oxymetholone-treated mice compared with the dexamethasone controls, which provides indirect evidence that FS exerts favorable and potent effects on muscle preservation (Table III).

Various toxic substances arising from lipid peroxidation destroy surrounding tissue (71), and oxidative stress is also an important inducer of muscle atrophy in both disuse and muscle cachexia (7). GSH is a representative endogenous antioxidant and prevents tissue damage by keeping ROS at low levels and at specific cellular concentrations and is recognized as a protective antioxidant factor in tissue (72). SOD is one of the antioxidant enzymes that contributes to enzymatic defense mechanisms, and CAT is an enzyme that catalyzes the conversion of H_2_O_2_ to H_2_O (73). The inhibition of the increase in lipid peroxidation and ROS levels, together with an increase in the GSH content and SOD and CAT activity in damaged muscle tissu is also important in terms of protecting muscle against atrophic changes (32,74). 4-HNE is an α,β-unsaturated hydroxyalkenal produced by lipid peroxidation in cells, and has been used as a valuable tissue lipid peroxidation marker. It is considered as a possible causal agent of numerous diseases, such as chronic inflammation, neurodegenerative diseases, adult respiratory distress syndrome, atherogenesis, diabetes and different types of cancer (75,76). Nitrotyrosine is a product of tyrosine nitration mediated by reactive nitrogen species, such as peroxynitrite anion and nitrogen dioxide. It is detected in large amounts under pathological conditions, and is considered a marker of iNOS-dependent, reactive nitrogen species-induced nitrative stress (77,78). In the present study, FS protected the gastrocnemius muscle against oxidative stress induced by dexamethasone in a dose-dependent manner, particularly the increase in lipid peroxidation and ROS formation, the decrease in the GSH content and SOD and CAT activity, and the increase in nitrotyrosine and 4-HNE in the muscle fibers (Tables IV and VI). Oxymetholone also showed potent antioxidant effects against the dexamethasone-induced depletion of antioxidant defense systems; this result is in accordance with previously published studies on anabolic steroids (79,80).

Muscle mass and structure are determined by the balance between protein degradation and synthesis (2). In the protein degradation pathway, ATP-ubiquitin-dependent proteolysis is the process most responsible for muscle wasting (81). Three enzymes are involved in the polyubiquitination cascades in this process: E1 (ubiquitin-activating), E2 (ubiquitin-conjugating) and E3 (ubiquitin ligase). It has recently been established that muscle-specific E3 ubiquitin ligases, such as atrogin-1 and MuRF1 play critical roles in muscle atrophy (2). Atrogin-1 contains an SCF complex (Skp, Cull and Roc1) (82) and directly interacts with calcineurin A and α-actinin-2 at the Z-disc (83). MuRF1 is a member of the RING finger-B-box-coiled-coil family (84) and interacts with titin at the M band (85). Previous studies have demonstrated that the expression levels of atrogin-1 and MuRF1 are increased in atrophic skeletal muscles and that mice deficient in either atrogin-1 or MuRF1 are resistant to muscle atrophy (3,82,86). In addition, a marked increase in atrogin-1 and MuRF1 mRNA expression levels has been detected in GLU-induced catabolic muscle atrophy (14). In the present study, a marked elevation in the gastrocnemius muscle atrogin-1 and MuRF1 mRNA expression levels was also observed in the dexamethasone controls compared with the intact vehicle controls. This increase in mRNA expression was inhibited by treatment with FS in a dose-dependent manner, providing direct evidence that FS exerts potent protective effects on muscle through the downregulation of atrogin-1 and MuRF1, which are involved in muscle protein degradation (Table V).

The insulin-like growth factor 1 (IGF-1)/PI3K/Akt signaling pathway is known to play a pivotal role in activating protein synthesis (2). PI3K, which is activated by insulin or IGF, in turn activates Akt, a serine/threonine kinase, which is located downstream of PI3K, phosphorylates glycogen synthase kinase-3β and mammalian target of rapamycin (mTOR), thereby inducing hypertrophy (6). As shown in Table V, a marked downregulation in the Akt1 and PI3K mRNA expression levels was observed in the mice in the dexamethasone control group, indicating catabolic muscle atrophic changes. However, FS upregulated the Akt1 and PI3K mRNA expression levels in a dose-dependent manner compared with the dexamethasone controls, providing direct evidence that FS promotes muscle protein synthesis and prevents dexamethasone-induced catabolic muscle atrophy, similar to the effects of oxymetholone.

Adenosine is known to modulate various physiological functions of the cardiovascular system and of most tissues, including skeletal muscle (87,88). The involvement of adenosine is proposed in both the regulation of blood flow to skeletal muscle (89) and in the synergistic effect of contraction and insulin-stimulated glucose uptake in skeletal muscle (90). The majority of the physiological effects of adenosine are believed to be mediated through specific adenosine receptors (91). Among these, adenosine A1R has shown cytoprotective effects on skeletal muscle (92). TRPV4, a member of the TRP channel superfamily (93,94), is a Ca^2+^-permeable non-selective cation channel that appears to play a mechanosensory or osmosensory role in several musculoskeletal tissue and prevents muscle atrophy or bone loss (94,95). In this study, dexamethasone significantly decreased the adenosine A1R and TRPV4 mRNA expression levels in gastrocnemius muscle due to proteolysis related to catabolic muscle atrophy. However, FS upregulated the mRNA expression levels of adenosine A1R and TRPV4 (involved in muscle growth) in a dose-dependent manner when compared with the dexamethasone controls (Table V), again providing direct evidence that FS increased muscle growth and resistance to dexamethasone-induced catabolic muscle atrophy to a similar extent as oxymetholone.

Myostatin, a secreted growth differentiation factor, is a member of the TGF-β protein family that inhibits muscle differentiation and growth in the process known as myogenesis. Myostatin is produced primarily in skeletal muscle cells, circulates in the blood and acts on muscle tissue by binding a cell-bound receptor called the activin type II receptor (96,97). Myostatin is a potent negative regulator of muscle growth (14,81). The sirtuin family of proteins possesses NAD^+^-dependent deacetylase activity and/or ADP ribosyltransferase activity. The 7 mammalian sirtuins, SIRT1-7, are localized differentially within cells and have a variety of functions. SIRT1 is the most extensively studied member of the family and regulates diverse biological processes ranging from cell proliferation, differentiation, apoptosis and metabolism (98). It controls the transcription of the peroxisome proliferator-activated receptor-γ co-activator 1 α in skeletal muscle (99) and induces cachexia by inhibiting muscle regeneration (100). In catabolic muscle atrophy, the mRNA expression levels of myostatin and SIRT1 have been detected along with a decrease in muscle mass (14,99), and this was also observed following treatment with dexamethasone in the present study. This increase in myostatin mRNA expression was inhibited by FS in a dose-dependent manner, and FS also inhibited the increase in the numbers of myostatin-immunoreactive fibers observed in immunohistochemical analysis in a dose-dependent manner, again providing direct evidence that FS exerts sufficiently potent muscle protective effects through the downregulation of myostatin and SIRT1 (Table V).

Catabolic muscle atrophic changes induced by GLUs include characteristic histopathological changes involving diminishing muscle fiber diameters, microvacuolation, collagen deposition and fibrosis, along with protein degradation (13,17), which were also observed in the present study. The histopatho-logical inhibition of muscle atrophic changes by treatment with FS or oxymetholone, demonstrated in this study, is considered valuable evidence that these substances can preserve denervation-related muscle atrophy (Fig. 6 and Table VI).

In conclusion, the results from the present study support a favorable ameliorating effect of FS on muscle atrophy induced by dexamethasone, by exerting anti-inflammatory and antioxidant effects related to muscle fiber protection that may be due to an increase in protein synthesis and a decrease in protein degradation. These effects of FS may help improve various muscle atrophies with various etiologies. The effects of treatment with 500 mg/kg FF were comparable to those obseved with treatment with 50 mg/kg oxymetholone, a 17α-alkylated anabolic-androgenic steroid, which has been used for the treatment of various muscle disorders.

## Figures and Tables

**Table I:** Oligonucleotide primers used for RT-qPCR in this study.

Target		Primer sequence (5′→3′)	Size (bp)	Gene ID
Atrogin-1	Forward	CAGCTTCGTGAGCGACCTC	244	67731
	Reverse	GGCAGTCGAGAAGTCCAGTC		
MuRF 1	Forward	GACAGTCGCATTTCAAAGCA	194	433766
	Reverse	GCCTAGCACTGACCTGGAAG		
PI3K p85α	Forward	GCCAGTGGTCATTTGTGTTG	236	18708
	Reverse	ACACAACCAGGGAAGTCCAG		
Akt1	Forward	ATGAACGACGTAGCCATTGTG	116	11651
	Reverse	TTGTAGCCAATAAAGGTGCCAT		
Adenosine A1R	Forward	TGTTCCCAGGGCCTTTCAC	155	11539
	Reverse	TAATGGACTGAGACTAGCTTGACTGGTA		
TRPV4	Forward	CAGGACCTCTGGAAGAGTGC	165	63873
	Reverse	AAGAGCTAGCCTGGACACCA		
Myostatin	Forward	CCTCCACTCCGGGAACTGA	185	17700
	Reverse	AAGAGCCATCACTGCTGTCATC		
SIRT1	Forward	TTCACATTGCATGTGTGTGG	175	93759
	Reverse	TGAGGCCCAGTGCTCTAACT		
18s Ribosomal RNA	Forward	AGCCTGAGAAACGGCTACC	252	19791
	Reverse	TCCCAAGATCCAACTACGAG		

MuRF1, muscle RING-finger protein-1; PI3k, phosphatidylinositol 3-kinase; A1R, adenosine A1 receptor; TRPV4, transient receptor potential cation cannel subfamily V member 4; SIRT1, sirtuin 1.

**Table II:** Primary antisera and detection kits used in this study.

Antisera or detection kits	Code	Source	Dilution
Primary antisera^a^			
Anti-4-Hydroxynonenal polyclonal antibody	Ab46545	Abcam, Cambridge, UK	1:100
Anti-Nitrotyrosine polyclonal antibody	06-284	Millipore Corporation, Billerica, CA, USA	1:200
Anti-nitric oxide synthase2 (N-20) polyclonal antibody	sc-651	Santa Cruz Biotechnology, Burlingame, CA, USA	1:100
Anti-GDF8/Myostatin antibody	Ab71808	Abcam, Cambridge, UK	1:50
Detection kits			
Vectastain Elite ABC kit	PK-6200	Vector Laboratories Inc., Burlingame, CA, USA	1:50
Peroxidase substrate kit	SK-4100	Vector Laboratories Inc., Burlingame, CA, USA	1:50

a All antisera were diluted by 0.01 M phosphate buffered saline.

**Table III:** Changes in serum biochemistry in the mice with dexamethasone-induced muscle atrophy.

Groups	Creatine serum levels (mg/dl)	CK serum levels (IU/l)	LDH serum levels (IU/l)
Controls			
Intact vehicle	0.34±0.05	79.75±19.91	566.63±135.65
Dexamethasone	0.76±0.11^a^	258.50±50.32^d^	144.25±50.47^d^
Reference			
Oxymetholone	0.45±0.11^b^,^c^	146.88±30.17^d^,^e^	330.38±84.23^d^,^e^
FS-treated mice			
500 mg/kg	0.46±0.12^b^,^c^	148.75±37.44^d^,^e^	319.25±108.50^d^,^e^
250 mg/kg	0.52±0.10^b^,^c^	178.38±28.81^d^,^e^	286.88±101.48^d^,^e^
125 mg/kg	0.60±0.12^b^,^c^	202.13±18.15^d^,^f^	230.75±41.82^d^,^e^

Values are expressed the means ± SD of 8 mice.

a p<0.01 and

b p<0.05 as compared with the intact vehicle control by the LSD test.

c p<0.01 as compared with dexamethasone control by LSD test.

d p<0.01 as compared with the intact vehicle control by the MW test.

e p<0.01 and

f p<0.05 as compared with the dexamethasone control by the MW test. CK, creatine kinase; LDH, lactate dehydrogenase; FS, *Fructus Schisandrae*.

**Table IV:** Changes in gastrocnemius muscle antioxidant defense systems in mice with dexamethasone-induced muscle atrophy.

Groups	Fundus antioxidant defense systems				
	MDA (nM/mg protein)	ROS (RFU/*μ*g protein)	GSH (nM/mg protein)	SOD (nM/mg protein)	CAT (U/mg protein)
Controls					
Intact vehicle	1.72±0.57	20.17±10.07	0.66±0.11	27.74±6.76	6.32±1.30
Dexamethasone	8.12±1.12^a^	61.22±10.95^a^	0.19±0.06^a^	11.64±1.46^e^	2.06±0.38^e^
Reference					
Oxymetholone	4.59±0.61^a^,^c^	35.76±11.06^a^,^c^	0.36±0.07^a^,^c^	19.39±3.19^e^,^f^	3.40±0.49^e^,^f^
FS-treated mice					
500 mg/kg	4.21±1.23^a^,^c^	34.01±11.69^b^,^c^	0.37±0.09^a^,^c^	20.93±2.18^e^,^f^	3.50±0.77^e^,^f^
250 mg/kg	5.33±1.17^a^,^c^	40.18±11.75^a^,^c^	0.31±0.07^a^,^c^	18.21±1.89^e^,^f^	3.22±0.50^e^,^f^
125 mg/kg	6.35±0.82^a^,^c^	44.82±8.53^a^,^c^	0.28±0.05^a^,^d^	16.54±2.54^e^,^f^	2.74±0.28^e^,^f^

Values are expressed as the means ± SD of 8 mice.

a p<0.01 and

b p<0.05 as compared with the intact vehicle control by the LSD test.

c p<0.01 and

d p<0.05 as compared with the dexamethasone control by the LSD test.

e p<0.01 as compared with the intact vehicle control by the MW test.

f p<0.01 as compared with the dexamethasone control by the MW test. MDA, malondialdehyde; ROS, reactive oxygen species; GSH, glutathione; SOD, superoxide dismutase; CAT, catalase; FS, *Fructus Schisandrae*.

**Table V:** Changes in gastrocnemius muscle mRNA expression in mice with dexamethasone-induced muscle atrophy.

Target	Controls		Reference	FS-treated mice (mg/kg)		
	Intact vehicle	Dexamethasone	Oxymetholone	500	250	125
Atrogin-1	1.02±0.13	4.77±0.82^e^	2.43±0.56^e^,^f^	2.41±0.43^e^,^f^	3.06±0.87^e^,^f^	3.36±0.98^e^,^g^
MuRF 1	0.98±0.07	5.80±1.16^e^	2.77±1.11^e^,^f^	2.33±0.61^e^,^f^	3.41±0.58^e^,^f^	4.17±1.21^e^,^g^
PI3K p85α	1.09±0.08	0.65±0.16^a^	1.23±0.44^c^	1.29±0.40^c^	1.07±0.33^c^	0.90±0.15
Akt 1	0.95±0.12	0.52±0.10^a^	0.83±0.11^b^,^c^	0.87±0.08^c^	0.78±0.16^a^,^c^	0.73±0.12^a^,^c^
Adenosine A1R	1.01±0.12	0.54±0.12^a^	0.90±0.07^b^,^c^	0.88±0.10^b^,^c^	0.78±0.09^a^,^c^	0.75±0.13^a^,^c^
TRPV4	0.99±0.12	0.41±0.12^a^	0.71±0.15^a^,^c^	0.74±0.11^a^,^c^	0.64±0.15^a^,^c^	0.57±0.12^a^,^d^
Myostatin	1.09±0.14	6.97±0.72^e^	3.22±1.36^e^,^f^	2.96±1.12^e^,^f^	3.89±0.99^e^,^f^	4.91±1.58^e^,^g^
SIRT1	1.10±0.21	8.65±3.70^e^	3.02±0.96^e^,^f^	2.85±0.60^e^,^f^	3.65±0.78^e^,^f^	4.43±0.72^e^,^f^

Values are expressed as the means ± SD of 8 mice, relative expression levels/18s ribosomal RNA.

a p<0.01 and

b p<0.05 as compared with the intact vehicle control by the LSD test.

c p<0.01 and

d p<0.05 as compared with the dexamethasone control by the LSD test.

e p<0.01 as compared with the intact vehicle control by the MW test.

f p<0.01 and

g p<0.05 as compared with the dexamethasone control by the MW test. PI3K, phosphatidylinositol 3-kinase; adenosine A1R, adenosine A1 receptor; TRPV4, transient receptor potential cation cannel subfamily V member 4; SIRT1, sirtuin 1; FS, *Fructus Schisandrae*.

**Table VI:** Histopathological and immunohistochemical anlaysis of the changes in gastrocnemius muscle in mice with dexamethasone-induced muscle atrophy.

Index	Controls		Reference	FS-treated mice (mg/kg)		
	Intact vehicle	Dexamethasone	Oxymetholone	500	250	125
General histomorphometry						
Fiber diameter (*μ*m)	50.16±5.62	24.01±5.13^a^	39.24±2.79^a^,^b^	39.84±3.80^a^,^b^	35.75±5.55^a^,^b^	30.16±2.81^a^,^b^
Collagen (%)	4.46±1.54	30.54±3.83^c^	18.15±1.92^c^,^d^	18.87±3.26^c^,^d^	20.84±2.44^c^,^d^	25.58±2.92^c^^e^
Immunohistomorphometry (fibers/mm^2^)						
Nitrotyrosine	6.25±1.49	68.25±12.90^c^	31.75±10.18^c^,^d^	31.00±5.55^c^,^d^	42.75±17.09^c^,^d^	50.88±11.69^c^^e^
4-HNE	4.38±1.51	70.75±6.71^a^	36.25±8.66^a^,^b^	37.88±15.75^a^,^b^	49.50±12.14^a^,^b^	54.50±13.15^a^,^b^
iNOS	7.38±2.77	46.50±5.29^a^	21.00±5.10^a^,^b^	29.50±4.17^a^,^b^	32.75±4.86^a^,^b^	36.75±7.65^a^,^b^
Myostatin	2.00±1.31	52.25±11.16^c^	25.38±10.50^c^,^d^	27.38±7.73^c^,^d^	34.88±7.26^c^,^d^	39.75±6.27^c^,^e^

Values are expressed as the means ± SD of 8 mice.

a p<0.01 as compared with the intact vehicle control by the LSD test.

b p<0.01 as compared with the dexamethasone control by the LSD test.

c p<0.01 as compared with the intact vehicle control bythe MW test.

d p<0.01 and

e p<0.05 as compared with the dexamethasone control by the MW test. 4-HNE, 4-hydroxynonenal; iNOS, inducible nitric oxide synthase FS, *Fructus Schisandrae*.
